# Repeatability and reproducibility of FreeSurfer, FSL-SIENAX and SPM brain volumetric measurements and the effect of lesion filling in multiple sclerosis

**DOI:** 10.1007/s00330-018-5710-x

**Published:** 2018-09-21

**Authors:** Chunjie Guo, Daniel Ferreira, Katarina Fink, Eric Westman, Tobias Granberg

**Affiliations:** 1 Division of Clinical Geriatrics, Department of Neurobiology, Care Sciences and Society, Karolinska Institutet, Stockholm, Sweden; 2 Department of Radiology, The First Hospital of Jilin University, Changchun, China; 3 Department of Clinical Neuroscience, Karolinska Institutet, Stockholm, Sweden; 4 Department of Neurology, Karolinska University Hospital, Stockholm, Sweden; 5 Division of Neuroradiology, Department of Radiology, Karolinska University Hospital, 141 86 Stockholm, Sweden

**Keywords:** Multiple sclerosis, Magnetic resonance imaging, Brain, Reproducibility of results, Imaging, three-dimensional

## Abstract

**Objectives:**

To compare the cross-sectional robustness of commonly used volumetric software and effects of lesion filling in multiple sclerosis (MS).

**Methods:**

Nine MS patients (six females; age 38±13 years, disease duration 7.3±5.2 years) were scanned twice with repositioning on three MRI scanners (Siemens Aera 1.5T, Avanto 1.5T, Trio 3.0T) the same day. Volumetric T_1_-weighted images were processed with FreeSurfer, FSL-SIENAX, SPM and SPM-CAT before and after 3D FLAIR lesion filling with LST. The whole-brain, grey matter (GM) and white matter (WM) volumes were calculated with and without normalisation to the intracranial volume or FSL-SIENAX scaling factor. Robustness was assessed using the coefficient of variation (CoV).

**Results:**

Variability in volumetrics was lower within than between scanners (CoV 0.17–0.96% vs. 0.65–5.0%, *p*<0.001). All software provided similarly robust segmentations of the brain volume on the same scanner (CoV 0.17–0.28%, *p*=0.076). Normalisation improved inter-scanner reproducibility in FreeSurfer and SPM-based methods, but the FSL-SIENAX scaling factor did not improve robustness. Generally, SPM-based methods produced the most consistent volumetrics, while FreeSurfer was more robust for WM volumes on different scanners. FreeSurfer had more robust normalised brain and GM volumes on different scanners than FSL-SIENAX (*p*=0.004). MS lesion filling changed the output of FSL-SIENAX, SPM and SPM-CAT but not FreeSurfer.

**Conclusions:**

Consistent use of the same scanner is essential and normalisation to the intracranial volume is recommended for multiple scanners. Based on robustness, SPM-based methods are particularly suitable for cross-sectional volumetry. FreeSurfer poses a suitable alternative with WM segmentations less sensitive to MS lesions.

**Key Points:**

*• The same scanner should be used for brain volumetry. If different scanners are used, the intracranial volume normalisation improves the FreeSurfer and SPM robustness (but not the FSL scaling factor).*

*• FreeSurfer, FSL and SPM all provide robust measures of the whole brain volume on the same MRI scanner. SPM-based methods overall provide the most robust segmentations (except white matter segmentations on different scanners where FreeSurfer is more robust).*

*• MS lesion filling with Lesion Segmentation Toolbox changes the output of FSL-SIENAX and SPM. FreeSurfer output is not affected by MS lesion filling since it already takes white matter hypointensities into account and is therefore particularly suitable for MS brain volumetry.*

**Electronic supplementary material:**

The online version of this article (10.1007/s00330-018-5710-x) contains supplementary material, which is available to authorized users.

## Introduction

Multiple sclerosis (MS) is a common chronic neuroinflammatory and neurodegenerative disease [1]. Demyelinating lesions in the brain and spinal cord are the pathological hallmarks of MS, which are detectable *in vivo* with magnetic resonance imaging (MRI). MRI has therefore become an essential tool for the diagnosis and monitoring of disease activity in MS [1, 2]. In MS, the lesion volume reflects the inflammatory burden while atrophy measures quantify neurodegenerative aspects of the disease, which play an important role in all disease stages [3]. Volumetry is therefore commonly used as a secondary endpoint in clinical trials [4]. Furthermore, volumetry can be helpful in improving our understanding of the disease since atrophy patterns have been shown to be different in MS compared to other demyelinating disorders [5].

Obtaining robust imaging biomarkers in MS for assessment of the inflammatory and neurodegenerative burden of disease is, however, challenging [3]. Brain volumetry is influenced by several subject-related factors such as hydration status, inflammation and clinical therapy [6]. MS lesions can specifically affect tissue segmentations since white matter (WM) lesions can be misclassified as grey matter (GM) or cerebrospinal fluid (CSF) [7, 8]. Brain volumetry is also impacted by technical factors such as MRI field strength and scanner model, as well as post-processing related issues [8–10]. Understanding the effect and magnitude of technical factors is important when planning MRI studies [8].

There are several freely available tools for automated brain volumetry that are commonly applied in MS. Popular choices include FreeSurfer [11], Structural Image Evaluation with Normalisation of Atrophy Cross-sectional (SIENAX) [12] and Statistical Parametric Mapping (SPM) [13]. These software can automatically pre-process and segment T_1_-weighted images of the brain. FreeSurfer is computationally demanding and is based on a combined volumetric- and surface-based segmentation aimed to reduce partial volume effects from the convoluted shape of the cortical ribbon [11]. FreeSurfer uses a template-driven approach to provide a detailed parcellation and segmentation of the cortex and subcortical structures. SIENAX, part of the FMRIB Software Library (FSL), is computationally less demanding but only provides measurements of the gross tissue volumes (WM, GM and CSF) [12]. FSL-SIENAX relies on registration to the Montreal Neurological Institute 152 template for skull stripping and then performs intensity-based segmentation; the template registration step provides a scaling factor that can be used for normalisation. SPM is based on non-linear registration of the brain to a template and segments brain tissues by assigning tissue probabilities per voxel [13]. Computational Anatomy Toolbox (SPM-CAT) is an extension for SPM that provides segmentations with a different segmentation approach based on spatial interpolation, denoising, additional affine registration steps, local intensity correction, adaptive segmentation and partial volume segmentation [14]. Like FSL-SIENAX, the SPM-based methods are less computationally demanding, relative to FreeSurfer, and only provide gross brain tissue volumes.

The primary purpose of this study was to compare the repeatability on the same scanner and the reproducibility on different scanners for brain tissue segmentations in FreeSurfer, FSL-SIENAX, SPM and SPM-CAT. A secondary aim was to study the effect of automated lesion filling to reduce MS lesion-related brain tissue segmentation bias.

## Materials and methods

### Participants

Nine MS patients (six females, three males; mean age 38±13 years; mean disease duration 7.3±5.2 years) diagnosed according to the McDonald 2010 diagnostic criteria [15], were prospectively recruited from the outpatient clinic at the Department of Neurology, Karolinska University Hospital in Huddinge, Stockholm, Sweden, among consecutive patients referred for a clinical MRI. The participants were representative of the MS population in Sweden, with all subtypes represented in proportion to their frequency in clinical practice: six relapsing-remitting (RR), two secondary progressive, one primary progressive [16]. Exclusion criteria were contraindications to MRI, neurological co-morbidities or a history of head trauma (none were excluded). The physical disability of the patients was assessed according to the Expanded Disability Status Scale [17] by an MS-experienced neurologist (K.F.). The median physical disability score was 2.0 (range 1.0–5.5). The study was approved by the local ethics committee and written informed consent was obtained from all participants.

### MRI protocol

**Fig. 1:**
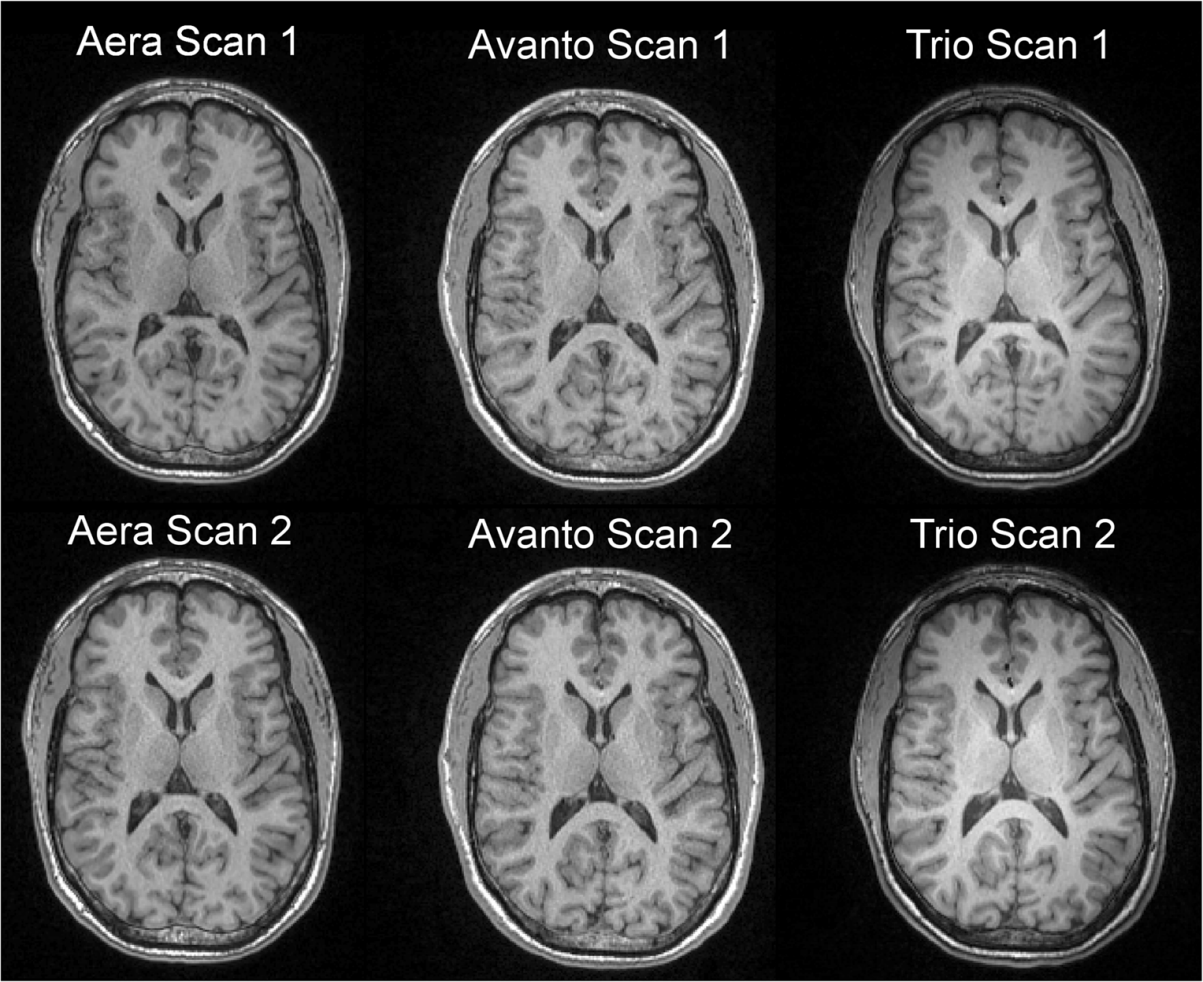
Example of mid-axial slices from the six 3D T_1_-weighted acquisitions in a 35-year-old male (referred to as MS1 in Online Supplementary Table 1) with relapsing-remitting multiple sclerosis and an Expanded Disability Status Scale score of 2.0. For each of the three scanners (Siemens Aera 1.5 T, Siemens Avanto 1.5 T and Siemens Trio 3.0 T) two acquisitions were made with repositioning in between

All participants were scanned twice on the same day on all three clinical MRI systems used in the study: Siemens Aera (1.5 T), Avanto (1.5 T) and Trio (3.0 T) (Siemens Healthcare, Erlangen, Germany). A 3D T_1_-weighted magnetisation-prepared rapid gradient-echo (MPRAGE) sequence was acquired twice with repositioning in between, resulting in a total of six T_1_-weighted volumes per participant. A representative example of the MPRAGE acquisitions is illustrated in Fig. 1. One 3D T_2_-weighted Fluid-Attenuated Inversion Recovery (FLAIR) was additionally acquired on each scanner for lesion segmentation. The MRI acquisition parameters are detailed in Table 1.

**Table 1:** MRI acquisition parameters

	Aera	Avanto	Trio
Field strength, T	1.5	1.5	3.0
3D MPRAGE			
Voxel size, mm^3^	1.0×1.0×1.5	1.0×1.0×1.5	1.0×1.0×1.5
Field-of-view, mm^2^	226×250	249×249	249×249
Repetition time, ms	1900	1900	1900
Inversion time, ms	1100	1100	900
Echo time, ms	3.02	3.55	3.39
Flip angle, °	15	15	9
Number of slices	160	160	160
3D FLAIR			
Voxel size, mm^3^	1.0×1.0×1.0	1.0×1.0×1.0	0.5×0.5×1.0
Field-of-view, mm^2^	227×260	227×260	250×250
Repetition time, ms	5000	6000	6000
Inversion time, ms	1800	2200	2100
Echo time, ms	333	333	388
Flip angle, °	120	120	120
Number of slices	176	176	160

*FLAIR* Fluid-Attenuated Inversion Recovery, *MPRAGE* Magnetisation-Prepared Rapid Gradient-Echo

### Image analysis

**Fig. 2:**
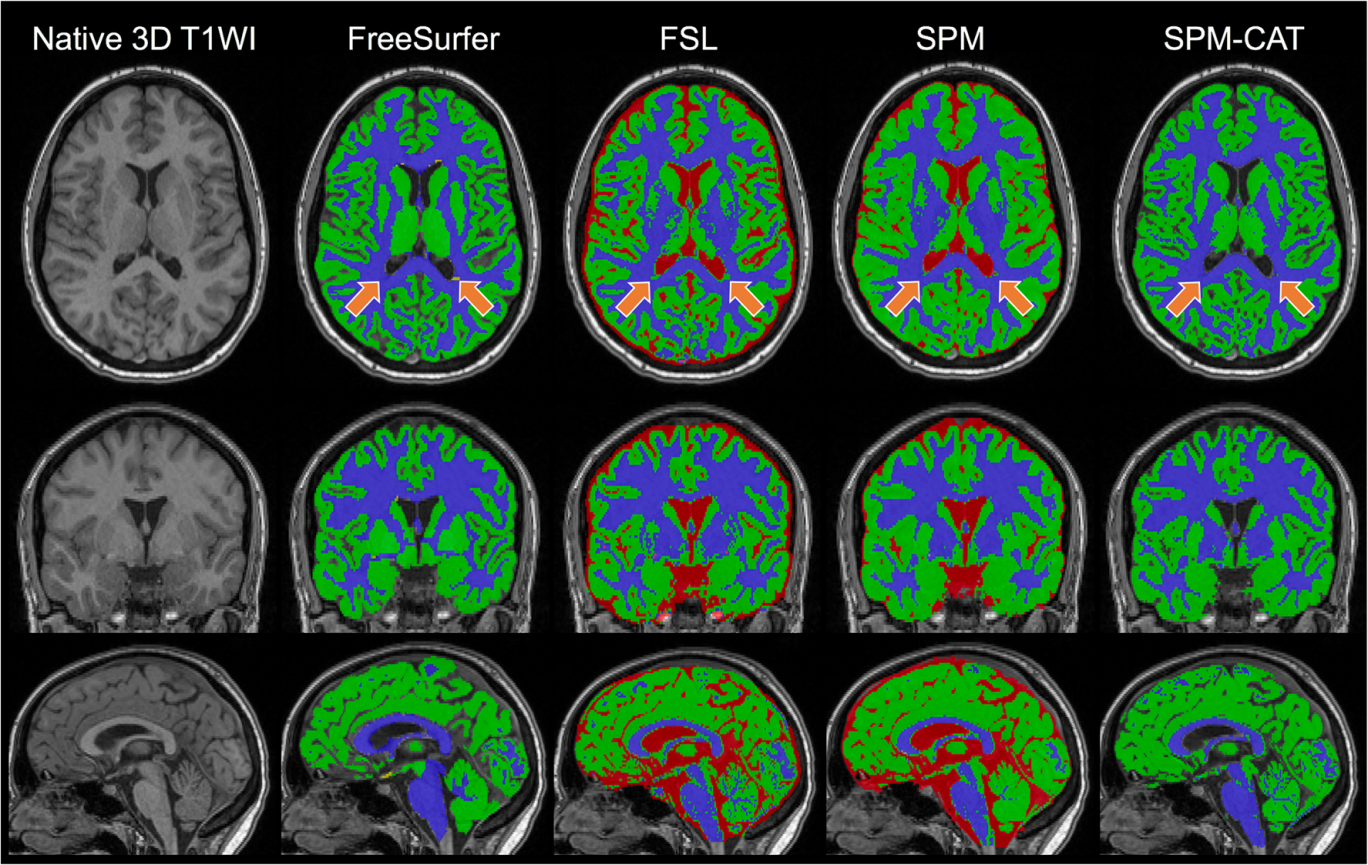
Volumetric segmentations in FreeSurfer 6.0.0, FSL-SIENAX 5.0, SPM 12 and SPM-CAT12 in a 20-year-old female (referred to as MS6 in Online Supplementary Table 1) with relapsing-remitting multiple sclerosis and an Expanded Disability Status Scale score of 1.0. Grey matter is displayed in green, white matter in blue and cerebrospinal fluid in red. The exemplified segmentations were based on the first scan on the Aera scanner for this participant, which was the scan with the lowest lesion volume (0.33 ml) in the study. Please note that FreeSurfer specifically segments white matter hypointensities (yellow), highlighted with orange arrows, and includes these in the brain volume, but not in the white matter volume. Meanwhile, FSL-SIENAX, SPM and SPM-CAT classify the white matter hypointensities as grey matter and/or cerebrospinal fluid (orange arrows). *CAT* Computational Anatomy Toolbox, *FSL-SIENAX* FMRIB Software Library Structural Image Evaluation with Normalisation of Atrophy Cross-sectional, *SPM* Statistical Parametric Mapping, *T1WI* T_1_-weighted imaging

Each of the six 3D T_1_-weighted volumes from each participant was analysed cross-sectionally and processed in FreeSurfer, FSL-SIENAX, SPM and SPM-CAT. No additional pre-processing or manual intervention was performed to avoid introducing biases in the tissue segmentations. All input and output underwent visual quality assurance by an experienced rater (T.G.) and were found to be of satisfactory quality. Examples of the volumetric output are presented in Fig. 2.

#### FreeSurfer

FreeSurfer 6.0.0 (http://surfer.nmr.mgh.harvard.edu, Harvard University, Boston, MA, USA) was used to perform automatic processing as previously described [11, 18]. FreeSurfer was run with the options ‘-mprage’ and for the 3.0 T data also ‘-3T’, as recommended by its developers. The variable ‘Brain Segmentation Volume Without Ventricles from Surf’ was used as the FreeSurfer estimation of the brain volume, which excludes the brainstem. The variable ‘Total grey matter volume’ was used as the estimation of the GM volume. The WM volume was assessed by summing the ‘cerebral WM’, ‘cerebellar WM’, ‘brainstem’ and ‘corpus callosum’ FreeSurfer variables. It is notable that FreeSurfer specifically segments white matter hypointensities. For normalisation purposes, the brain volume, GM volume and WM volume were divided by the ‘Estimated Total Intracranial Volume’.

#### FSL-SIENAX

The SIENAX method implemented in FSL 5.0 (https://fsl.fmrib.ox.ac.uk/fsl/fslwiki/SIENA, Oxford University, Oxford, UK) was used to obtain an automated quantification of the brain volume, GM volume and WM volume with automatic normalisation for head size with a subject-specific scaling factor, as previously described [19]. For this study, FSL-SIENAX was run with the optimised brain extraction parameters ‘-B -f 0.1’, in accordance with previous recommendations for MS studies [20].

#### SPM

Statistical Parametric Mapping, SPM12, (http://www.fil.ion.ucl.ac.uk/spm, University College London, London, UK) was used to automatically obtain the GM volume, WM volume and total intracranial CSF volume according to an adapted workflow as previously described [21]. The segment tool was run using the default settings. The brain volume in SPM was defined as the sum of the GM and WM volumes. For normalisation, the intracranial volume was used, which was calculated by summing the GM, WM and CSF volumes.

#### SPM-CAT

The Computational Anatomy Toolbox (CAT) 12 is an extension to SPM12 (http://www.neuro.uni-jena.de/cat/index.html, Jena University Hospital, Jena, Germany) [14]. The cross-sectional data segmentation tool was run using the default settings. The brain volume in SPM-CAT was defined as the sum of the GM and WM volumes and the total intracranial volume was used for normalisation.

#### Lesion filling

**Fig. 3:**
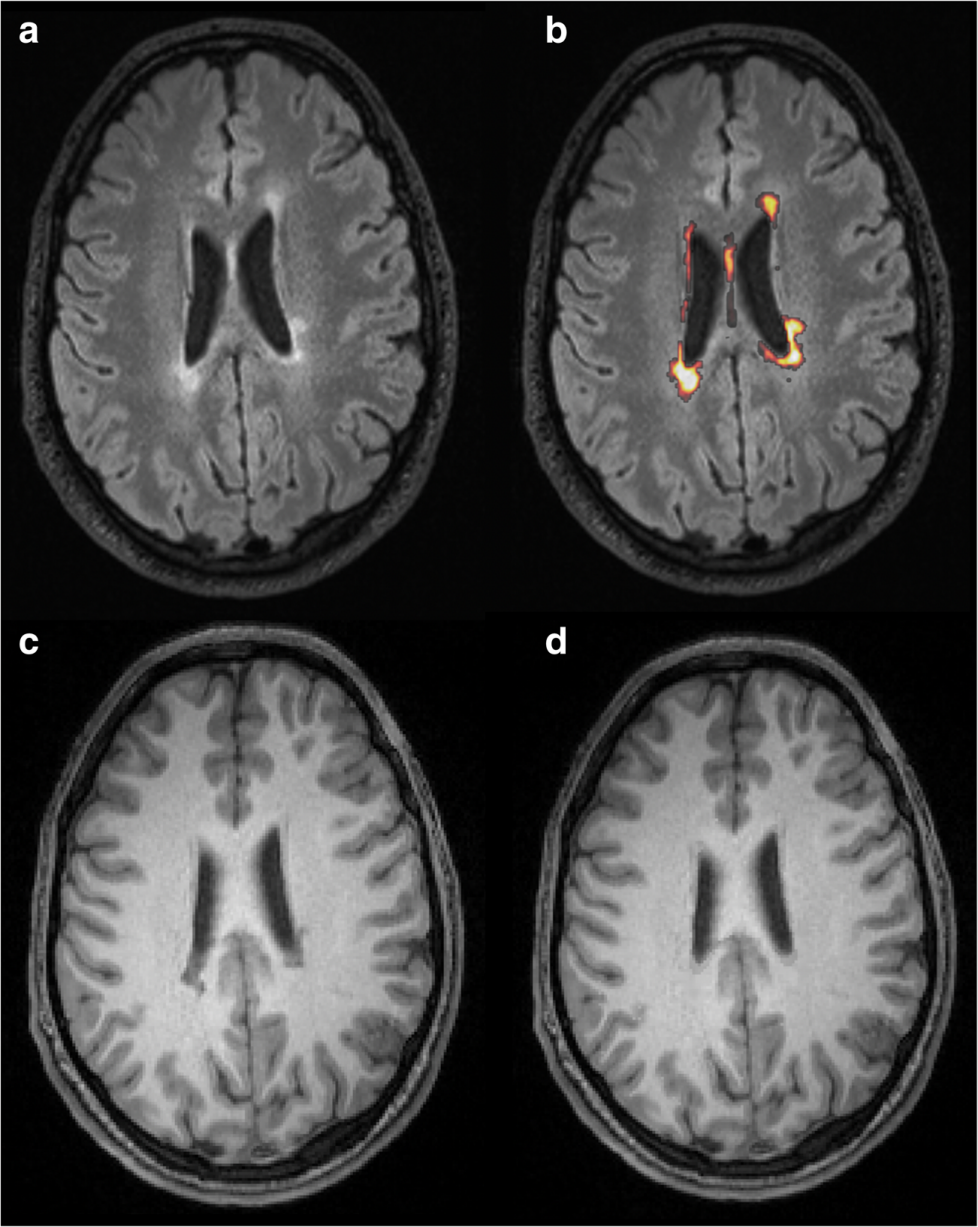
Illustration of the lesion segmentation and filling procedure in a 34-year-old male (referred to as MS5 in Online Supplementary Table 1) with relapsing-remitting multiple sclerosis and an Expanded Disability Status Scale score of 1.5. This representative scan from the Siemens Trio 3.0 T scanner provided the median lesion volume of the cohort (1.8 ml). The 3D T_2_-weighted FLAIR image (**a**) was used for lesion segmentation in Lesion Segmentation Toolbox, resulting in a probabilistic lesion mask (**b**, displayed as a heat map overlaid on **a**). The lesion mask was used to fill in lesions on the 3D T_1_-weighted image (**c**), providing the lesion-filled 3D T_1_-weighted image (**d**)

Lesion filling was performed on all 3D FLAIR volumes in SPM12 using the lesion probability algorithm in Lesion Segmentation Toolbox 2.0.10 (LST, http://www.applied-statistics.de/lst.html,Technische Universität München, Munich, Germany) [22]. LST provides an automated probabilistic lesion segmentation, specifically developed for MS. It also provides automatic lesion filling without the need for parameter optimisation or binary thresholding of the lesion masks. The FLAIR lesion probability maps were used to perform lesion filling on the corresponding T_1_-weighted volumes from the same scanner [22]. Figure 3 illustrates the input and output of the lesion filling procedure.

### Statistical analysis

SPSS Statistics 24.0 was used for the statistical analysis (IBM Corporation, Armonk, NY, USA). Due to the limited sample size, the data were treated as non-parametric. The robustness of repeated measures was assessed using the within-subject coefficient of variation (CoV). For intra-scanner repeatability, the measurements from the first and the second scan from the same scanner were used: CoV_Intra-scanner_ = SD/mean of Scan 1 and Scan 2. For the inter-scanner reproducibility, the first scans from each of the three scanners were used: CoV_Inter-scanner_ = SD/mean of Scan 1_Aera_, Scan 1_Avanto_, Scan 1_Trio_. Paired comparisons were tested using the Wilcoxon signed ranks test with two-tailed exact significance. Group comparisons between the four software were tested using the Friedman test and in case of significant differences among the software, post hoc paired analyses were performed with the Wilcoxon signed ranks test. Correction for multiple comparisons was performed using the Benjamini-Hochberg procedure separately for the intra-scanner CoVs, inter-scanner CoVs and for each Friedman test post hoc analysis [23]. A corrected *p*<0.05 was considered statistically significant. All reported *p*-values were significant after correction for multiple comparisons, unless otherwise specified.

## Results

### Comparability of the brain volumetry from different software

There were notable differences in the numeric brain tissue segmentation output from FreeSurfer, SIENAX, SPM and SPM-CAT, as detailed in Table 2. A full report of the volumetric output can be found in Online Supplementary Table 1.

**Table 2:** Brain tissue volumes with/without normalisation and with/without lesion filling

	FreeSurfer		FSL-SIENAX		SPM		SPM-CAT	
	Original	Lesion-filled	Original	Lesion-filled	Original	Lesion-filled	Original	Lesion-filled
Brain volume	1223±101	1222±94.0; *p*=0.83	1300±117	1299±117; *p*<0.001	1213±94.0	1217±94.0; *p*<0.001	1211±97.0	1212±98.0; *p*<0.001
WM volume	558±60.1	557±58.6; *p*=0.63	642±71.6	643±71.1; *p*=0.015	451±43.0	457±39.6; *p*<0.001	534±46.0	535±54.0; *p*<0.001
GM volume	684±71.8	681±70.5; *p*=0.67	658±52.3	658±56.7; *p*<0.001	783±85.2	786±85.4; *p*=0.74	672±81.0	671±83.0; *p*<0.001
Normalised brain volume	72.2±3.7	72.0±4.0; *p*=0.46	1582±84.0	1576±84.0; *p*=0.34	78.4±7.9	78.5±7.9; *p*<0.001	76.1±9.0	76.2±8.9; *p*<0.001
Normalised WM volume	32.5±1.2	32.4±1.4; *p*=0.43	785±38.8	782±30.1; *p*=0.039^*^	29.5±2.9	29.5±3.0; *p*<0.001	33.5±3.7	33.7±3.4; *p*<0.001
Normalised GM volume	40.8±3.6	40.9±3.7; *p*=0.92	782±109	783±112; *p*=0.013	48.7±6.1	48.6±6.2; *p*=0.50	41.7±6.4	41.5±6.3; *p*<0.001

All metrics given as median±interquartile range. Non-normalised (upper three rows) and FSL-SIENAX measurement are given in millilitres. Normalised measurements of FreeSurfer and SPM are given as unit-less tissue fractions in %. *P*-values represent the comparison of original and lesioned-filled volumes by Wilcoxon signed ranks test (exact significance, two-tailed)

*CAT* Computational Anatomy Toolbox, *FSL-SIENAX* FMRIB Software Library Structural Image Evaluation with Normalisation of Atrophy Cross-sectional, *GM* Grey matter, *SPM* Statistical Parametric Mapping, *WM* White matter

^*^Not statistically significant after correction for multiple comparisons

### Repeatability and reproducibility of non-normalised brain volumetry

Repeated measurements on the same scanner generally resulted in lower variability than measurements on the different scanners (median CoV 0.17–0.96% vs. 0.65–5.0%, *p*<0.001 by Wilcoxon signed ranks test), as further detailed in Table 3. Overall, the brain volume was the most robust tissue segmentation within scanners, with the lowest variability (median CoV 0.17–0.28%), and a comparable performance of all segmentation methods (*p*=0.076 by Friedman test). For all other volumetrics there were, however, differences between the software, both for the intra-scanner repeatability (WM volume *p*=0.017, GM volume *p*=0.004, normalised brain volume *p*=0.012, normalised WM volume *p*<0.001 and normalised GM volume *p*=0.004) and the inter-scanner reproducibility (brain volume *p*=0.002, WM volume *p*<0.001, GM volume *p*<0.001, normalised brain volume *p*<0.001, normalised WM volume *p*=0.007 and normalised GM volume *p*=0.001), all by the Friedman test. Post hoc analyses with corrections for multiple comparisons showed that the SPM-based methods generally had the lowest CoV of the four software, reflecting good repeatability and reproducibility, with the exception of WM segmentations on different scanners, where FreeSurfer was more robust. The two SPM methods performed similarly in most regards, with the exception of inter-scanner WM segmentations where SPM-CAT had significantly lower variability.

**Table 3:** Repeatability and reproducibility of the brain tissue volumes

		FS	FSL	SPM	SPM-CAT	FS vs. FSL	FS vs. SPM	FS vs. SPM-CAT	FSL vs. SPM	FSL vs. SPM-CAT	SPM vs. SPM-CAT
Intra-scanner CoV	Brain volume	0.28±0.23	0.17±0.84	0.17±0.24	0.19±0.30	-	-	-	-	-	-
	WM volume	0.96±0.90	0.48±0.72	0.24±0.47	0.41±0.47	*p*=0.27	*p*=0.002	*p*=0.005	*p*=0.034	*p*=0.14	*p*=0.67
	GM volume	0.75±0.95	0.47±0.73	0.23±0.44	0.31±0.42	*p*=0.75	*p*=0.004	*p*=0.013	*p*<0.001	*p*=0.003	*p*=0.47
	Normalised brain volume	0.26±0.27; *p*=0.19	0.40±0.66; *p*<0.001	0.20±0.23; *p*=0.29	0.18±0.28; *p*=0.62	*p*=0.004	*p*=0.63	*p*=0.40	*p*=0.019	*p*=0.008	*p*=0.66
	Normalised WM volume	0.92±0.83; *p*=0.59	0.49±0.86; *p*=0.14	0.27±0.53; *p*=0.46	0.43±0.49; *p*=0.80	*p*=0.46	*p*<0.001	*p*=0.008	*p*<0.001	*p*=0.046^*^	*p*=0.29
	Normalised GM volume	0.59±0.88; *p*=0.79	0.50±1.1; *p*=0.041^*^	0.24±0.51; *p*=0.99	0.28±0.32; *p*=0.80	*p*=0.49	*p*=0.004	*p*=0.025	*p*=0.013	*p*=0.014	*p*=0.29
Inter-scanner CoV	Brain volume	2.7±0.49	2.8±0.45	2.3±0.65	2.3±0.60	*p*=0.82	*p*=0.004	*p*=0.004	*p*=0.055	*p*=0.027	*p*=0.91
	WM volume	1.9±1.5	2.5±1.3	5.0±0.98	3.5±1.1	*p*=0.055	*p*=0.004	*p*=0.020	*p*=0.012	*p*=0.25	*p*=0.004
	GM volume	2.8±1.1	3.9±3.3	1.1±1.1	1.5±1.2	*p*=0.20	*p*=0.004	*p*=0.008	*p*=0.004	*p*=0.004	*p*=0.30
	Normalised brain volume	0.65±0.64; *p*=0.004	2.6±2.5; *p*=0.82	1.1±0.75; *p*=0.004	1.0±0.54; *p*=0.004	*p*=0.004	*p*=0.50	*p*=0.13	*p*=0.004	*p*=0.012	*p*=0.65
	Normalised WM volume	1.8±2.1; *p*=0.30	2.7±4.5; *p*=0.50	4.7±1.6; *p*=0.004	2.4±1.6; *p*=0.004	*p*=0.039*	*p*=0.008	*p*=0.25	*p*=1.0	*p*=0.16	*p*=0.004
	Normalised GM volume	0.65±0.58; *p*=0.004	3.3±4.1; *p*=0.36	1.2±0.96; *p*=1.0	1.4±0.85; *p*=0.36	*p*=0.004	*p*=0.074	*p*=0.16	*p*=0.055	*p*=0.004	*p*=0.91

*P*-values for the normalised volumes represent the comparison of the coefficient of variation with the non-normalised volumes. All pairwise comparisons by Wilcoxon signed ranks test (exact significance, two-tailed)

*CoV* Coefficient of variation, *CAT* Computational Anatomy Toolbox, *FS* FreeSurfer, *FSL* FMRIB Software Library, *GM* Grey matter, *SPM* Statistical Parametric Mapping, *WM* White matter

^*^Not statistically significant after correction for multiple comparisons

### Effects of normalisation on brain volumetry

Normalising the brain tissue volumes did not have a statistically significant positive effect on the intra-scanner repeatability, as further detailed in Table 3. On the contrary, for the FSL-SIENAX normalised brain volume there was a worsening of the intra-scanner repeatability after normalisation with the scaling factor. Normalisation to the FSL-SIENAX scaling factor did not significantly improve the inter-scanner reproducibility either. In contrast, normalisation to the intracranial volume often improved the reproducibility between scanners for FreeSurfer and the SPM methods. Specifically, significant improvements in the reproducibility were seen for the FreeSurfer normalised brain volume and normalised grey matter volume as well as for the normalised brain volume and white matter volume for both SPM-based methods. When normalising the tissues, FreeSurfer became more robust than FSL-SIENAX across scanners for both the normalised brain volume and normalised GM volume.

### Effects of MS lesion filling

The median WM lesion volume was 1.8 ml (range 0.33–24 ml). There was no statistically significant effect of lesion filling on the FreeSurfer volumes, as detailed in Table 2. However, lesion filling caused changes in volumetrics from FSL-SIENAX, SPM and SPM-CAT. Most notably, highly significant changes were seen for all tissue compartments in SPM-CAT with increases in the estimations of the brain and WM volumes and decreases in the GM estimations, both for the non-normalised and normalised data. Lesion filling did not significantly affect the inter-scanner CoV for any of the software (data not shown).

## Discussion

We present a prospective head-to-head comparison of the robustness of four of the most popular freely available brain segmentation tools in a representative real-life MS cohort scanned twice on three different scanners on the same day. New versions of the tested software have recently been released. An important contribution of the current study is therefore that we provide an up-to-date evaluation of the intra- and inter-scanner variability of brain tissue measurements in MS, facilitating an appropriate choice of software for volumetric studies.

We found that the volumetric output differed between the software, which is expected since they have large technical differences [11–13]. Previous studies of earlier versions of the software have indeed also found significant differences in the output, both numerically and topographically [24–26]. While most previous studies have focused on differences and similarities in the segmentation results [24–26], the current study mainly focused on the robustness of the segmentation tools. Overall, we report that the variability in volumetrics was lower on the same scanner than between scanners, supporting recommendations to follow individuals on the same scanner [27, 28]. Although brain atrophy rates can be double that of normal aging in untreated MS patients [29], treated MS patients have atrophy rates around 0.5%/year [30]. To accurately capture atrophy rates, it is therefore important to have a variability lower than that. Our reported CoVs for intra-scanner (0.17–0.92%) and inter-scanner (0.65–5.0%) variability suggest that measurements are feasible within 1–2 years for the most robust methods on the same scanner. In contrast, several years need to pass to be able to capture atrophy on different scanners, even with normalisation.

SPM-based methods overall had the best repeatability and reproducibility of the four software (except WM segmentations where FreeSurfer was more robust) and are therefore particularly suitable for cross-sectional MS studies. This is in line with a previous international study of two MS patients scanned at multiple sites and a segmentation challenge in persons with diabetes mellitus and cardiovascular risk factors [31, 32]. We also found that the whole-brain volume was the most robust volumetric, consistent with previous results [31, 33]. This could be explained by lower variability with a large volume of interest and a larger contrast difference of CSF versus brain parenchyma compared to GM/WM segmentations. In studies with differences in the MRI protocols, it can therefore be recommended to primarily focus on the brain volume. Interestingly, there was no significant difference in the intra-scanner robustness of the software for the brain volume, meaning that all studied software can be favoured for cross-sectional MS studies of the brain volume.

The current study focuses on some of the most commonly used freely available automated segmentation tools for brain volumetrics in MS, but there are several other segmentation tools available, such as AFNI and BrainSuite. While we provide information on the robustness of the studied software, the choice of software must also take other factors into account, such as which types of images are available, user skills and technical requirements [8]. In this study, we only provided the T_1_-weighted images for segmentation, which is the only image contrast that FSL-SIENAX and SPM-CAT are optimised for [12, 14]. Previous results with segmentation based on multiple contrasts or multi-parametric maps have shown especially good robustness [32–34]. Evaluating such approaches is therefore an interesting avenue for future studies. From a technical standpoint, full functionality of SPM requires a MATLAB license [13], but a standalone version of SPM or FreeSurfer could be suitable alternatives since FreeSurfer was found to provide more robust normalised measurements between scanners than FSL-SIENAX, consistent with previous results [35]. While FreeSurfer is computationally more intense than the other software, it also provides more detailed regional morphometry.

Normalisation of the brain volumetrics to the intracranial volume generally improved the comparability of results between scanners, in line with previous recommendations [8]. This is likely due to a reduction of scaling effects between scanners [8]. However, using the scaling factor in FSL-SIENAX did not improve the robustness, suggesting that such normalisation may not be sufficient. Overall, there was also a lack of improvement in the repeatability within scanners for all three software with the normalisation. This finding likely reflects that normalisation procedures are less critical if measurements are produced on the same scanner. In clinical practice and longitudinal studies it is, however, important to consider that the variability in measurements are likely to be higher than that presented in this study, where all measurements were performed on the same day [31].

In terms of the effect of MS lesion filling, we found that lesion filling affected the volumetric results mainly for SPM and SPM-CAT, but also for FSL-SIENAX. These results are consistent with a previous MS study showing increased accuracy of SPM8 segmentations after lesion filling [36]. Of note, no effect was seen on the FreeSurfer volumes with lesion filling, likely due to the fact that FreeSurfer specifically segments WM T_1_-hypointensities and thus take these into account during the WM segmentations [11].

This study has some limitations. First, the sample size is small, but in total there were 54 measurements since each patient was scanned twice on three scanners and the study showed statistically significant differences in robustness of the software. Second, the MRI scanners were all from the same manufacturer, while higher inter-scanner variability would be expected with multiple vendors [31]. Third, although the results of the study could change by adjusting acquisition or processing parameters, these results reflect the standard procedures for MRI in MS at Karolinska University Hospital and we used recommended post-processing options [11, 13, 20]. There was a difference in the resolution between the FLAIR volumes, which could affect the lesion filling but this difference was consistent for the input of all software. Lastly, the current study focused solely on cross-sectional segmentation methods while the robustness of segmentations can be improved by including a priori knowledge of several time-points [19, 35, 37]. We therefore recommend future studies to also focus on comparing the robustness of longitudinal segmentation methods.

In conclusion, the results highlight the importance of consistently using the same scanner and normalising to the intracranial volume when multiple scanners are used. The output from FreeSurfer, FSL-SIENAX and SPM differ but all three software provide cross-sectional brain volume segmentations with similar intra-scanner robustness. SPM-based methods overall produced the most consistent results, while FreeSurfer had less variability in WM volume segmentations across scanners and was less affected by WM lesions.

## Electronic supplementary material

Supplementary Table 1 (XLSX 60 kb)

## Abbreviations

CoV: Coefficient of variation
CSF: Cerebrospinal fluid
FLAIR: Fluid-attenuated inversion recovery
FSL: Functional magnetic resonance imaging of the brain software library
GM: Grey matter
LST: Lesion segmentation toolbox
MS: Multiple sclerosis
SIENAX: Structural image evaluation with normalisation of atrophy cross-sectional
SPM: Statistical parametric mapping
WM: White matter

## References

[1] Thompson AJ, Banwell BL, Barkhof F (2018). Diagnosis of multiple sclerosis: 2017 revisions of the McDonald criteria. Lancet Neurol.

[2] Filippi M, Rocca MA, Ciccarelli O (2016). MRI criteria for the diagnosis of multiple sclerosis: MAGNIMS consensus guidelines. Lancet Neurol.

[3] Enzinger C, Barkhof F, Ciccarelli O (2015). Nonconventional MRI and microstructural cerebral changes in multiple sclerosis. Nat Rev Neurol.

[4] Barkhof F, Calabresi PA, Miller DH, Reingold SC (2009). Imaging outcomes for neuroprotection and repair in multiple sclerosis trials. Nat Rev Neurol.

[5] Liu Y, Duan Y, Huang J (2018). Different patterns of longitudinal brain and spinal cord changes and their associations with disability progression in NMO and MS. Eur Radiol.

[6] Sampat MP, Healy BC, Meier DS, Dell'Oglio E, Liguori M, Guttmann CR (2010) Disease modeling in multiple sclerosis: assessment and quantification of sources of variability in brain parenchymal fraction measurements. Neuroimage 52:1367–1373. 10.1016/j.neuroimage.2010.03.075 10.1016/j.neuroimage.2010.03.075 20362675

[7] Chard DT, Jackson JS, Miller DH, Wheeler-Kingshott CAM (2010). Reducing the impact of white matter lesions on automated measures of brain gray and white matter volumes. J Magn Reson Imaging.

[8] Vrenken H, Jenkinson M, Horsfield MA (2013). Recommendations to improve imaging and analysis of brain lesion load and atrophy in longitudinal studies of multiple sclerosis. J Neurol.

[9] Han X, Jovicich J, Salat D (2006). Reliability of MRI-derived measurements of human cerebral cortical thickness: the effects of field strength, scanner upgrade and manufacturer. Neuroimage.

[10] Jovicich J, Czanner S, Han X (2009). MRI-derived measurements of human subcortical, ventricular and intracranial brain volumes: reliability effects of scan sessions, acquisition sequences, data analyses, scanner upgrade, scanner vendors and field strengths. Neuroimage.

[11] Fischl B (2012). FreeSurfer. Neuroimage.

[12] Jenkinson M, Beckmann CF, Behrens TE, Woolrich MW, Smith SM (2012) FSL. Neuroimage 62:782–790. 10.1016/j.neuroimage.2011.09.015 10.1016/j.neuroimage.2011.09.015 21979382

[13] Ashburner J, Friston KJ (2005). Unified segmentation. Neuroimage.

[14] Gaser C, Dahnke R (2016) CAT - a computational anatomy toolbox for the analysis of structural MRI data. p 1 10.1093/gigascience/giae049 PMC11299546 39102518

[15] Polman CH, Reingold SC, Banwell B (2011). Diagnostic criteria for multiple sclerosis: 2010 revisions to the McDonald criteria. Ann Neurol.

[16] Lublin FD, Reingold SC, Cohen JA (2014). Defining the clinical course of multiple sclerosis: the 2013 revisions. Neurology.

[17] Kurtzke JF (1983). Rating neurologic impairment in multiple sclerosis an expanded disability status scale (EDSS). Neurology.

[18] Ferreira D, Voevodskaya O, Imrell K (2014). Multiple sclerosis patients lacking oligoclonal bands in the cerebrospinal fluid have less global and regional brain atrophy. J Neuroimmunol.

[19] Smith SM, Zhang Y, Jenkinson M (2002). Accurate, robust, and automated longitudinal and cross-sectional brain change analysis. Neuroimage.

[20] Popescu V, Battaglini M, Hoogstrate WS (2012). Optimizing parameter choice for FSL-Brain Extraction Tool (BET) on 3D T1 images in multiple sclerosis. Neuroimage.

[21] Ashburner J (2007). A fast diffeomorphic image registration algorithm. Neuroimage.

[22] Schmidt P, Gaser C, Arsic M (2012). An automated tool for detection of FLAIR-hyperintense white-matter lesions in multiple sclerosis. Neuroimage.

[23] Benjamini Y, Hochberg Y (1995). Controlling the false discovery rate: a practical and powerful approach to multiple testing. J R Stat Soc Series B Stat Methodol.

[24] Klauschen F, Goldman A, Barra V, Meyer-Lindenberg A, Lundervold A (2009) Evaluation of automated brain MR image segmentation and volumetry methods. Hum Brain Mapp 30:1310–1327. 10.1002/hbm.20599 10.1002/hbm.20599 PMC6870639 18537111

[25] Heinen R, Bouvy WH, Mendrik AM, Viergever MA, Biessels GJ, de Bresser J (2016) Robustness of automated methods for brain volume measurements across different MRI field strengths. PLoS One 11:e0165719. 10.1371/journal.pone.0165719 10.1371/journal.pone.0165719 PMC5087903 27798694

[26] Kazemi K, Noorizadeh N (2014). Quantitative comparison of SPM, FSL, and brainsuite for brain MR image segmentation. J Biomed Phys Eng.

[27] Wattjes MP, Rovira À, Miller D et al (2015) Evidence-based guidelines: MAGNIMS consensus guidelines on the use of MRI in multiple sclerosis—establishing disease prognosis and monitoring patients. Nat Rev Neurol 11:597–606. 10.1038/nrneurol.2015.157 10.1038/nrneurol.2015.157 26369511

[28] Vågberg M, Axelsson M, Birgander R (2017). Guidelines for the use of magnetic resonance imaging in diagnosing and monitoring the treatment of multiple sclerosis: recommendations of the Swedish Multiple Sclerosis Association and the Swedish Neuroradiological Society. Acta Neurol Scand.

[29] De Stefano N, Giorgio A, Battaglini M (2010). Assessing brain atrophy rates in a large population of untreated multiple sclerosis subtypes. Neurology.

[30] De Stefano N, Stromillo ML, Giorgio A et al (2015) Establishing pathological cut-offs of brain atrophy rates in multiple sclerosis. J Neurol Neurosurg Psychiatry:jnnp-2014-309903. 10.1136/jnnp-2014-309903 10.1136/jnnp-2014-309903 PMC4717444 25904813

[31] Biberacher V, Schmidt P, Keshavan A (2016). Intra- and interscanner variability of magnetic resonance imaging based volumetry in multiple sclerosis. Neuroimage.

[32] Mendrik AM, Vincken KL, Kuijf HJ et al (2015) MRBrainS challenge: online evaluation framework for brain image segmentation in 3T MRI scans. In: Computational intelligence and neuroscience. https://www.hindawi.com/journals/cin/2015/813696/. Accessed 11 Jul 2018 10.1155/2015/813696 PMC4680055 26759553

[33] Granberg T, Uppman M, Hashim F (2016). Clinical feasibility of synthetic mri in multiple sclerosis: a diagnostic and volumetric validation study. AJNR Am J Neuroradiol.

[34] West J, Warntjes JB, Lundberg P (2011). Novel whole brain segmentation and volume estimation using quantitative MRI. Eur Radiol.

[35] Durand-Dubief F, Belaroussi B, Armspach JP et al (2012) Reliability of longitudinal brain volume loss measurements between 2 sites in patients with multiple sclerosis: comparison of 7 quantification techniques. AJNR Am J Neuroradiol. 10.3174/ajnr.A3107 10.3174/ajnr.A3107 PMC7964600 22790248

[36] Valverde S, Oliver A, Roura E (2015). Quantifying brain tissue volume in multiple sclerosis with automated lesion segmentation and filling. Neuroimage Clin.

[37] Reuter M, Schmansky NJ, Rosas HD, Fischl B (2012). Within-subject template estimation for unbiased longitudinal image analysis. Neuroimage.

